# Quantifying the relationship between arboviral infection prevalence and human mobility patterns among participants of the Communities Organized to Prevent Arboviruses cohort (COPA) in southern Puerto Rico

**DOI:** 10.1371/journal.pntd.0011840

**Published:** 2023-12-15

**Authors:** Maile T. Phillips, Liliana Sánchez-González, Talya Shragai, Dania M. Rodriguez, Chelsea G. Major, Michael A. Johansson, Vanessa Rivera-Amill, Gabriela Paz-Bailey, Laura E. Adams

**Affiliations:** 1 Dengue Branch, Division of Vector-borne Diseases, Centers for Disease Control and Prevention, San Juan, Puerto Rico; 2 Global Immunization Division, Center for Global Health, Centers for Disease Control and Prevention, Atlanta, Georgia, United States of America; 3 Ponce Health Sciences University/ Ponce Research Institute, Ponce, Puerto Rico; Australian Red Cross Lifelood, AUSTRALIA

## Abstract

Human movement is increasingly being recognized as a major driver of arbovirus risk and dissemination. The Communities Organized to Prevent Arboviruses (COPA) study is a cohort in southern Puerto Rico to measure arboviral prevalence, evaluate interventions, and collect mobility data. To quantify the relationship between arboviral prevalence and human mobility patterns, we fit multilevel logistic regression models to estimate odds ratios for mobility-related predictors of positive chikungunya IgG or Zika IgM test results collected from COPA, assuming mobility data does not change substantially from year to year. From May 8, 2018–June 8, 2019, 39% of the 1,845 active participants during the study period had a positive arboviral seroprevalence result. Most (74%) participants reported spending five or more weekly hours outside of their home. A 1% increase in weekly hours spent outside the home was associated with a 4% (95% confidence interval (CI): 2–7%) decrease in the odds of testing positive for arbovirus. After adjusting for age and whether a person had air conditioning (AC) at home, any time spent in a work location was protective against arbovirus infection (32% decrease, CI: 9–49%). In fact, there was a general decreased prevalence for individuals who visited locations that were inside and had AC or screens, regardless of the type of location (32% decrease, CI: 12–47%). In this population, the protective characteristics of locations visited appear to be the most important driver of the relationship between mobility and arboviral prevalence. This relationship indicates that not all mobility is the same, with elements like screens and AC providing protection in some locations. These findings highlight the general importance of AC and screens, which are known to be protective against mosquitoes and mosquito-transmitted diseases.

## Author summary

We characterized the relationship between arboviral infection prevalence and human mobility patterns among 1,845 participants in the Communities Organized to Prevent Arboviruses (COPA) study in Ponce, Puerto Rico. Among participants, 39% had a positive arboviral seroprevalence result. The majority (74%) reported spending 5 or more weekly hours outside of their home of residence. In general, more time spent outside the home was associated with decreased arboviral infection prevalence in this population. Arboviral prevalence was associated with the types of locations participants visited regularly, and characteristics of those locations. Work and locations with air conditioning (AC) or screens were protective for arboviral prevalence. Different from other studies, in this population, the characteristics of locations visited appear to be the most important drivers of the relationship between mobility and arboviral prevalence. This relationship indicates that not all mobility is the same, with elements like screens and AC providing protection in some locations. Previous studies have shown that household AC and screens are important factors in determining arboviral risk; however, results from this study further highlight their importance in non-residential locations where people visit. These findings support the use of screens and AC in all locations in areas at risk for arbovirus transmission.

*The findings and conclusions in this report are those of the authors and do not necessarily represent the official position of the U.S. Centers for Disease Control and Prevention*.

## Introduction

Dengue, Zika, and chikungunya viruses (DENV, ZIKV, CHIKV, respectively) are mosquito-transmitted viruses that present an increasing public health challenge in tropical regions [1,2]. Infections can be asymptomatic or produce mild to severe symptoms and long-term complications in infected individuals [2,3]. All three have endemic distribution throughout the tropics, although DENV is more widespread globally. An estimated 3.83 (95% confidence interval (CI): 3.45–4.09) billion people (roughly half of the global population) currently live in areas suitable for DENV transmission. The vast majority of the population impacted is located in Asia, followed by Africa and the Americas [3]. CHIKV and ZIKV emerged in the Western Hemisphere in the last decade and remain endemic, with the Americas still accounting for the largest percentage of the global burden of CHIKV and ZIKV [4].

DENV, ZIKV, and CHIKV have all impacted Puerto Rico in the past decade. DENV transmission is seasonal, with large epidemics roughly every four to five years [5]. The last dengue epidemic occurred in 2012 and 2013, with over 18,000 suspected and 9,200 confirmed dengue cases [6–8]. A chikungunya outbreak occurred in Puerto Rico in 2014 (over 4,500 confirmed cases), followed by a Zika outbreak in 2016 (over 36,000 confirmed cases) [9–12]. Together, these three arboviral diseases impose a considerable economic and health burden to the population, driving efforts to prevent and control transmission.

Multiple entomologic, behavioral, and environmental approaches have been implemented to control the primary vector of these arboviruses, *Aedes aegypti*, yet none have proven to be both effective and sustainable [13]. While *Ae*. *aegypti* mosquitoes generally move very little [14], human movement is increasingly being recognized as a major driver of arbovirus risk and dissemination. One study found that geographic locations with greater utilization of public transit, and hence mobility, was associated with greater dengue incidence [15]. A longitudinal DENV cohort study in Iquitos, Peru, found that human movement was an important factor in determining DENV risk, incidence patterns, and virus spread [16]. On a broader geographic scale, DENV transmission modeling using outbreak data from Pakistan showed that human mobility as measured by mobile phone data could predict the spread and timing of epidemics at a regional and national level [17]. While mobile phone data can illuminate many mobility patterns, they are not always available or accessible.

The impact of human mobility on arbovirus infection risk is an important consideration for the effectiveness of localized intervention measures such as mosquito control around homes. For example, a trial in Puerto Rico found that *Ae*. *aegypti* traps reduced CHIKV transmission in two communities, but that residents spending more time outside of those communities had higher infection risk than their neighbors [18]. Risk from outside communities can therefore reduce the impact of local control efforts.

In this study, we used data from a community cohort in Ponce, Puerto Rico to characterize human mobility patterns and identify differences in mobility by age, sex, and other demographic factors. Here, we define mobility patterns by the amount of time spent in non-resident locations and characteristics of those locations. We aimed to quantify the relationship between the prevalence of arboviral infection and weekly mobility patterns, including the types and characteristics of locations visited, to better inform interventions.

## Methods

### Study design

The Communities Organized to Prevent Arboviruses (COPA) cohort study design and data collection have been described in detail elsewhere [19]. COPA study objectives include determining incidence of arboviral infections, assessing burden and etiology of acute febrile illness, and evaluating the effectiveness of vector control methods. Briefly, COPA is an ongoing cohort study implemented in 2018 and includes approximately 4,000 participants and 2,200 households in Ponce, Puerto Rico. All of Ponce is considered at risk for DENV, CHIKV, and ZIKV. Inclusion criteria for study participation comprise individuals between 1–50 years of age who sleep in the residence four or more nights per week and have no plans to move in the next six months. Study participants are visited once a year after enrollment; new participants are offered enrollment to replace dropouts annually. Here, we examine data from the annual visits at baseline and in the second year.

**Fig 1:**
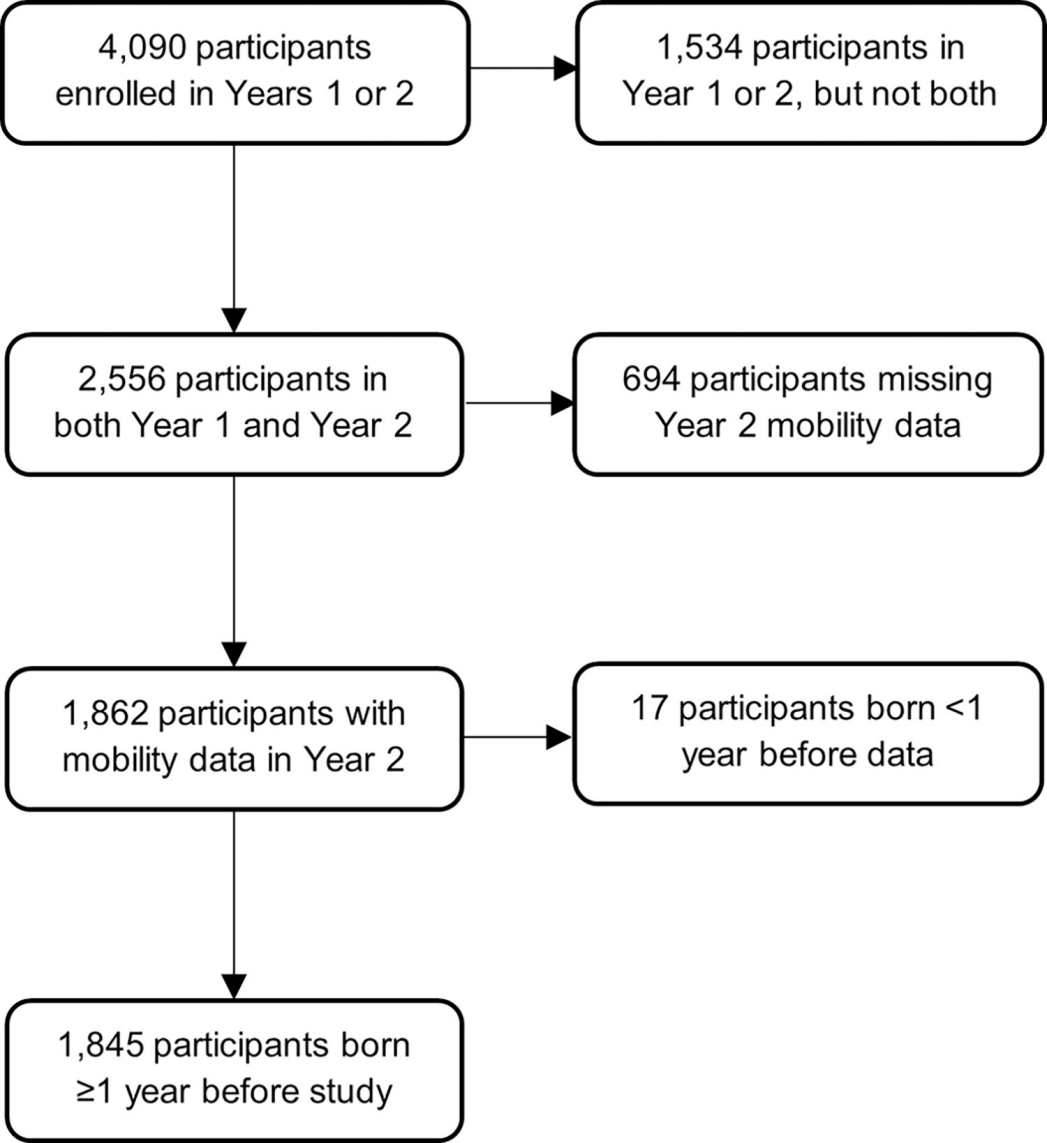
Flowchart diagram for inclusion of study population for mobility analysis.

For this analysis, the population was restricted to individuals who participated in COPA Year 2 during September 25, 2019–November 7, 2020, as a more comprehensive set of mobility questions was implemented during this period. We also limited the study population to participants who had arbovirus seroprevalence assessed in Year 1 (May 8, 2018–June 8, 2019), had household coordinates, who were born at least a year before the study period began, and who answered all mobility questions. With these exclusions, the dataset included 1,845 individuals (Fig 1).

### Seroprevalence data

All cohort participants provided serum samples collected by phlebotomy at annual visits for arbovirus serology testing. These laboratory methods have been described in detail elsewhere [19]. In Year 1, seroprevalence data were collected for CHIKV and ZIKV. Seropositivity was defined as a positive result for CHIKV IgG or ZIKV IgM. Of note, ZIKV IgM levels can remain positive for months to years post-infection [20]. ZIKV IgG testing was not available at the time of analysis, due to cross-reactivity of existing assays with DENV antibodies. To restrict the population to individuals who were alive during the time period when ZIKV circulation was at high levels in Puerto Rico (2016), individuals born after May 8, 2017 (less than 5% of the population) were excluded from the study population. Starting in Year 2, only DENV IgM seroprevalence data were collected. Due to low rates of DENV infections among COPA participants during the study period, consistent with local surveillance data, this study focuses on CHIKV and ZIKV as seroprevalence outcomes.

### Questionnaires and mobility data

Each study year, COPA participants answer questions about the household (income, presence of screens or air conditioning), individual demographics (age, sex, employment, education), history of febrile illness, and behaviors surrounding mosquito prevention. Individuals 14 years and older self-report, while parents answered for their children under 14 years of age. In this analysis, household, demographic, febrile history, and behavioral data were used from Year 2 to align with the mobility data.

In Year 2 of the study, an additional mobility-specific survey was administered at the same time as the general questionnaires (S1 Fig). Mobility questions included general measures (hours spent outside the home during a typical week; a continuous variable), and more specific metrics about other locations visited for more than 5 hours per week (types of locations, characteristics of locations, duration of time typically spent in those locations, and address and coordinates of locations). As detailed information on mobility was not collected in Year 1 when serology testing for CHIKV and ZIKV were performed, this analysis is restricted individuals with data available from both years with the assumption that people’s mobility on average does not drastically change from year to year. Since part of Year 2 coincided with several regional earthquakes and the COVID-19 pandemic, mobility questions were specified to ask about typical or usual behavior.

### Statistical analyses

To first examine differences in mobility by demographics and household characteristics, we fit multilevel linear regression models for each demographic variable separately as a predictor of mobility (log-transformed typical weekly hours spent outside the home). Due to similarities between household members, each model controlled for household clustering with a household-level (random effect) intercept. Adjusting for household clustering eliminated the need to further adjust for spatial correlation, which we verified by calculating Moran’s I with and without the household effect.

For the main analysis, we fit multilevel logistic regression models that controlled for household clustering to calculate unadjusted and adjusted odds ratios (ORs and aORs, respectively) for mobility-related predictors of a positive CHIKV IgG or ZIKV IgM result:

$$\mathrm{logit}\left({arbovirus}_{i}\right)=\beta_{0,i}+b_{1}mobility_{i}+\sum_{j}b_{j}X_{j,i}$$

where intercept *β*_0,*i*_ is a household-specific intercept (random effect) that controls for household clustering, exponentiated fixed slope *b*_1_ is the odds ratio of arboviral prevalence of previous infection (positive seroprevalence for CHIKV or ZIKV, referred to as prevalence below) for a one-unit increase in the mobility variable, and exponentiated fixed slope *b*_*j*_ is the odds ratio of arboviral prevalence for a one-unit increase in the selected demographic adjustment variable *j*.

Mobility variables included (log-transformed) typical weekly hours spent outside the home, specific geographic (neighborhood-level) locations people visited, types of locations participants visited (and how many weekly hours they spent there), and characteristics of destinations participants visited (and how many weekly hours they spent there). Information about the locations participants visited were limited to places where participants typically spent 5 or more hours per week. Options for types of locations included work (primary, secondary, tertiary), school, university, daycare, sports-related, recreational, gym, church, supermarket, other (non-supermarket) store, another house, or other. Too few participants reported spending 5+ weekly hours in many of the types of locations to analyze them separately, so we grouped similar categories together: sports, gym, or recreational; daycare, school, or university; or any type of work. Less than 5 participants reported spending 5 hours or more at church, another house, supermarket, other store, or other locations, and as a result these variables were excluded from location type analyses. Characteristics of locations visited included whether the location was indoors or outdoors, and whether the location had AC or screens. To examine the relationship with one of the recommended guidelines for mosquito protection, we created an additional combined variable for whether participants spent time in indoor locations with screens or AC.

We did not include any models with multiple mobility variables because they were highly correlated. Instead, we fit several models for each mobility variable separately. The adjusting demographic variables were selected as the combination of variables that minimized the Deviance Information Criterion (DIC) for each separate mobility variable. The final mobility model was chosen as the one with the lowest overall DIC compared to other models with the same mobility predictor.

We conducted the same analyses with CHIKV and ZIKV seroprevalence alone as outcomes and found no substantial differences compared to models with the combined outcome. To increase the sample size and to be able to generalize to arboviral prevalence, all final analyses used both CHIKV and ZIKV.

All analyses were performed using R version 1.4.1717 [21]. Models were fit using the R package *lme4* [22].

### Ethics statement

The COPA study was reviewed by the Centers for Disease Control and Prevention Human Subjects Office and approved by the Ponce Medical School Foundation, Inc. Institutional Review Board, approval number 171110-VR. COPA participants provided written consent for all annual study activities. All minor participants under 20 years of age had written consent from their parents/guardians. Additionally, children aged 14–20 provided their own written consent, and children aged 7–13 provided verbal consent.

## Results

Among 1,845 COPA participants that met inclusion criteria in this analysis, 39% had a positive arboviral antibody result. Approximately a fourth were positive for CHIKV only (24%), while less (8%) had positive results for ZIKV only or both viruses (7%). The median age in Year 2 was 32 (range: 4–52) years and 60% were female. Approximately 42% of participants lived in a household with income <$10,000, about a fourth were employed full-time (24%) and a third were students (34%), (Table 1). Most participants reported the presence of screens on windows or doors (68%) and the use of air conditioning (AC; 73%) in their home of residence.

**Table 1:** Demographic and household characteristics of COPA participants by arboviral (CHIKV and ZIKV) serostatus, Ponce, Puerto Rico, 2018–2020. Summary statistics are shown for the demographic variables in the study, stratified by arboviral infection result (Arbovirus–or +). All variables shown are categorical, and are presented with frequencies (column percentages). Min = Minimum; Max = Maximum.

	Arbovirus—(N = 1,131)	Arbovirus + (N = 714)	Overall (N = 1,845)
**Sex**			
*Female*	683 (60%)	431 (60%)	1114 (60%)
*Male*	448 (40%)	283 (40%)	731 (40%)
**Age category (years)**			
*0–10*	121 (11%)	30 (4%)	151 (8%)
*11–20*	251 (22%)	157 (22%)	408 (22%)
*21–30*	190 (17%)	130 (18%)	320 (17%)
*31–40*	208 (18%)	166 (23%)	374 (20%)
*41–50*	322 (28%)	203 (28%)	525 (28%)
*51+*	39 (3%)	28 (4%)	67 (4%)
**Household income (USD $)**			
*< $10*,*000*	431 (38%)	344 (48%)	775 (42%)
*$10*,*000 - $19*,*999*	198 (18%)	137 (19%)	335 (18%)
*$20*,*000 - $29*,*999*	153 (14%)	94 (13%)	247 (13%)
*$30*,*000 - $39*,*999*	119 (11%)	48 (7%)	167 (9%)
*$40*,*000 - $49*,*999*	68 (6%)	28 (4%)	96 (5%)
≥ *$50*,*000*	116 (10%)	29 (4%)	145 (8%)
*No response*	46 (4%)	34 (5%)	80 (4%)
**Employment**			
*Full-time work*	284 (25%)	154 (22%)	438 (24%)
*Part-time or informal work*	134 (12%)	86 (12%)	220 (12%)
*Student*	411 (36%)	209 (29%)	620 (34%)
*Stay at home person*	112 (10%)	124 (17%)	236 (13%)
*Retired or cannot work due to health issues*	30 (3%)	13 (2%)	43 (2%)
*Unemployed*	115 (10%)	106 (15%)	221 (12%)
*Other or no response*	45 (4%)	22 (3%)	67 (4%)
**Highest Level of Education Completed**			
*Grades 1 to 5*	281 (25%)	226 (32%)	507 (27%)
*Grades 6 to 8*	103 (9%)	33 (5%)	136 (7%)
*Grades 9 to 11*	90 (8%)	66 (9%)	156 (8%)
*Finished high school/GED*	89 (8%)	70 (10%)	159 (9%)
*Technical/Associate degree*	187 (17%)	139 (19%)	326 (18%)
*Bachelor’s degree*	223 (20%)	111 (16%)	334 (18%)
*Professional title or post-graduate studies*	89 (8%)	34 (5%)	123 (7%)
*No schooling*, *special education*, *or no response*	69 (6%)	35 (5%)	104 (6%)
**Air conditioning in the household**			
*No*	277 (24%)	221 (31%)	498 (27%)
*Yes*	854 (76%)	493 (69%)	1347 (73%)
**Screens in the household**			
*No*	283 (25%)	305 (43%)	588 (32%)
*Yes*	848 (75%)	409 (57%)	1257 (68%)

Participants typically spent a median of 35 (range: 0–152) weekly hours outside of their home. The majority (75%) of participants reported spending five or more weekly hours outside of their home, but only a small percentage (6%) reported spending five or more weekly hours outside of Ponce municipality. Individuals mostly spent time outside the home at work (28%); daycare, school, or university (26%); or sports, gym, or recreational activities (8%). A little less than half (45%) of the total population reported that they spent five or more weekly hours outside of the home in one or more indoor locations with screens or AC. When stratifying these mobility variables by whether participants were seropositive for CHIKV or ZIKV, the largest differences appeared to occur with weekly hours spent outside the home; time spent at daycare, school, or university; time spent at work; and time spent inside with AC or screens (Table 2).

**Table 2:** Mobility-related characteristics of COPA participants by arboviral (CHIKV and ZIKV) serostatus, Ponce, Puerto Rico, 2018–2020. Summary statistics are shown for the main mobility-related variables in the study, stratified by arboviral infection result (Arbovirus–or +). Continuous variables are presented with medians [Minimum, Maximum], while categorical variables are presented with frequencies (column percentages). Min = Minimum; Max = Maximum.

	Arbovirus—(N = 1,131)	Arbovirus + (N = 714)	Overall (N = 1,845)
**Weekly hours spent outside of home of residence**			
*Median [Min*, *Max]*	35 [0,152]	30 [0,114]	35 [0,152]
**5+ weekly hours spent outside of home of residence**			
*No*	264 (23%)	206 (29%)	470 (25%)
*Yes*	867 (77%)	508 (71%)	1375 (75%)
**5+ hours spent outside Ponce**			
*No*	1055 (93%)	675 (95%)	1730 (94%)
*Yes*	76 (7%)	39 (5%)	115 (6%)
**5+ weekly hours spent at work**			
*No*	812 (72%)	561 (79%)	1373 (74%)
*Yes*	319 (28%)	153 (21%)	472 (26%)
**5+ weekly hours spent at daycare, school, or university**			
*No*	796 (70%)	527 (74%)	1323 (72%)
*Yes*	335 (30%)	187 (26%)	522 (28%)
**5+ weekly hours spent at sports, gym, or recreational activities**			
*No*	1042 (92%)	651 (91%)	1693 (92%)
*Yes*	89 (8%)	63 (9%)	152 (8%)
**5+ weekly hours spent at an indoor location outside the home with screens or AC**			
*No*	586 (52%)	429 (60%)	1015 (55%)
*Yes*	545 (48%)	285 (40%)	830 (45%)

### Demographic factors associated with mobility

Age, sex, income, employment, and education were all single independent predictors of (log-transformed) weekly hours spent outside the home. Older age groups and less than full time employment were associated with decreased mobility (weekly hours spent outside the home), while male sex, higher household income and higher education were associated with increased mobility (Table 3, Single predictor [unadjusted] models).

**Table 3:** Results of unadjusted and multivariable linear regression models with demographic predictors of (log-transformed) hours spent outside the home. Demographic predictor coefficients of mobility (hours spent outside the home) are shown for the demographic variables (age group, sex, income, employment, and education) for the single-predictor regression models (left column) and best-fit multivariable model (right column). Note that age group and sex were not included in the best-fit multivariable model. Statistically significant estimates (α-level 0.05) are noted with an asterisk (*) in the “Sig.” column. Ref. = reference level; Coef. = coefficient; Lower/Upper = lower and upper bounds of 95% confidence interval; Sig. = statistically significant.

	Single predictor (unadjusted) models				Full (multivariable) model			
	*Coef*.	*Lower*	*Upper*	*Sig*.	*Coef*.	*Lower*	*Upper*	*Sig*.
	**MODEL 1**							
**Age Group (years; ref. <11)**								
*11 to 20*	-0.68	-1.42	0.07		--	--	--	
*21 to 30*	-0.49	-1.28	0.30		--	--	--	
*31 to 40*	-0.88	-1.61	-0.15	*	--	--	--	
*41 to 50*	-1.03	-1.77	-0.30	*	--	--	--	
*51+*	-1.04	-2.25	0.17		--	--	--	
	**MODEL 2**							
**Sex (ref. Female)**								
*Male*	0.84	0.47	1.20	*	--	--	--	
	**MODEL 3**				**MODEL 6**			
**Income (2019 USD; ref. <$10,000)**								
*$10*,*000 - $19*,*999*	1.75	1.07	2.42	*	0.94	0.30	1.57	*
*$20*,*000 - $29*,*999*	2.51	1.75	3.26	*	1.09	0.37	1.81	*
*$30*,*000 - $39*,*999*	2.47	1.59	3.35	*	0.85	0.01	1.69	*
*$40*,*000 - $49*,*999*	2.60	1.52	3.67	*	0.78	-0.24	1.80	
≥ *$50*,*000*	3.01	2.11	3.91	*	1.35	0.46	2.24	*
*No response*	0.79	-0.35	1.93		0.01	-1.03	1.07	
	**MODEL 4**							
**Employment (ref. full time)**								
*Part-time or informal work*	-0.74	-1.36	-0.12	*	-0.43	-1.07	0.20	
*Student*	-1.64	-2.10	-1.19	*	-0.72	-1.34	-0.11	*
*Stay at home person*	-4.45	-5.04	-3.85	*	-3.85	-4.50	-3.21	*
*Retired or cannot work due to health issues*	-4.62	-5.82	-3.43	*	-4.24	-5.44	-3.04	*
*Unemployed*	-4.59	-5.22	-3.96	*	-4.12	-4.78	-3.45	*
*Other or no response*	-4.38	-5.35	-3.41	*	-2.85	-4.02	-1.69	*
	**MODEL 5**							
**Education (ref. finished high school/GED)**								
*1 to 5*	1.20	0.45	1.94	*	-0.02	-0.78	0.74	
*6 to 8*	-0.55	-1.25	0.15		-1.35	-2.05	-0.65	*
*9 to 11*	-0.04	-0.73	0.65		-0.64	-1.31	0.02	
*Technical/Associate degree*	0.40	-0.18	0.97		0.20	-0.34	0.74	
*Bachelor’s degree*	1.48	0.90	2.05	*	0.49	-0.08	1.07	
*Professional title or post-graduate studies*	1.09	0.27	1.91	*	-0.38	-1.19	0.42	
*No schooling*, *special education*, *or no response*	-1.50	-2.36	-0.64	*	-1.69	-2.65	-0.75	*

Time spent in locations outside of the home of residence was generally lower for individuals 11 years and older compared to children less than 11 years of age (Table 3, Model 1). Compared to those who worked full-time, participants who typically carried out part-time or informal work, were students or homemakers, could not work due to health issues, were retired, or were unemployed were associated with lower levels of mobility (Table 3, Model 4).

Compared to females, males were associated higher mobility (Table 3, Model 2). Any household income level higher than $10,000 per year was associated with higher mobility compared to those with lower income (Table 3, Model 3).

The best-fit demographic multivariable model to predict weekly hours spent outside the home was the model with income, employment, and education (Table 3, Model 6). In this model, less than full-time employment was again associated with decreased mobility after adjusting for income and education, though the decrease was slightly less than when considered independently. Higher income was also associated with increased mobility after adjusting for other variables with slightly smaller impacts. Only those with no response to educational level and those who had finished grades 6–8 had a statistically significant estimate, but the overall variable still improved model fit.

### Mobility predictors of arboviral prevalence

**Fig 2:**
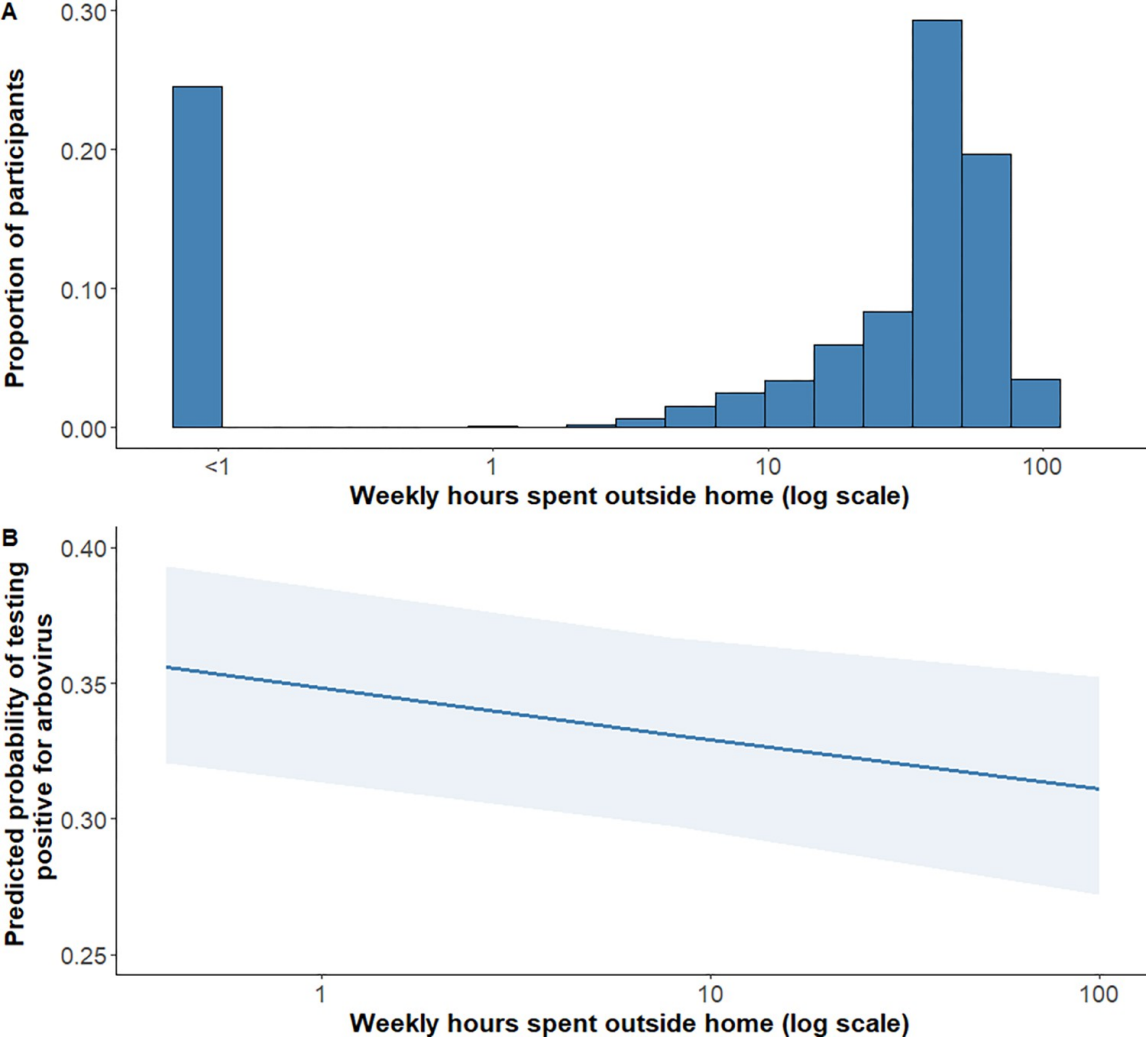
Proportion of participants and predicted probability of testing positive for arbovirus by typical (log-transformed) weekly hours spent outside the home. The proportion of participants (panel A) and the estimated probability of testing positive for arbovirus, after adjusting for age and screens or air conditioning presence in the home (panel B), are shown compared to the reported (log-transformed) typical weekly hours spent outside of the home of residence. In panel A, the proportion individuals who reported 0 weekly hours outside the home are noted in the bar with <1 weekly hours spent outside the home, due to the logarithmic scale of the data and axis. In panel B, the solid blue line represents the mean fixed effects predicted probability and the light blue band denotes the 95% confidence interval.

In general, more time spent outside the home was associated with decreased arboviral infection prevalence in this population (Fig 2). Either with or without adjusting for age group and AC or screens in the home, a 1% increase in weekly hours spent outside the home was associated with an estimated 0.04% (0.01–0.06%) decrease in the odds of testing positive for an arbovirus (Table 4, Models 7A and 7B). For example, a participant who typically spent 40 weekly hours outside the home would have an estimated 5% (1–9%) lower prevalence of arboviral infection compared to a participant who typically spent only 10 weekly hours outside the home. This decreased arboviral prevalence with increased mobility was not associated with travel to specific geographic neighborhoods and municipalities.

**Table 4:** Hierarchical logistic regression models of mobility variables to predict arboviral prevalence. Results of the hierarchical logistic regression models with statistically significant single predictors are shown. Column A shows the results from the single mobility predictor models, while column B shows the full adjusted model with the same mobility variable, adjusted for demographic variables (chosen as the models with the lowest Deviance Information Criterion value). Model 7 contains the mobility variable log-transformed weekly hours spent outside the home; Model 8 contains the mobility variable spending any amount of time over 5 hours at a work location (binary); Model 9 contains the mobility variable number of weekly hours (5 or more) spent at work; Model 10 contains the mobility variable spending any amount of time over 5 hours at a location indoors with air conditioning or screens (binary); and Model 11 contains the mobility variable number of weekly hours (5 or more) spent at a location indoors with air conditioning or screens. Statistically significant (confidence interval does not contain 1) coefficients are denoted with an asterisk (*). For estimates and/or confidence intervals that round to the null value (1), three or four significant digits are shown instead of two. OR = odds ratio; aOR = adjusted odds ratio; Ref. = reference level; log = log-transformed; AC = air conditioning; Lower/Upper = lower and upper bounds of 95% confidence interval; Sig. = statistically significant.

	A. Single predictor (unadjusted) models				B. Full (adjusted) model			
Variable	*OR*	*Lower*	*Upper*	*Sig*.	*aOR*	*Lower*	*Upper*	*Sig*.
**MODEL 7**								
*(Intercept)*	0.57	0.49	0.66	*	0.39	0.21	0.74	*
**Mobility variable**								
*log(hours outside home)*	0.96	0.93	0.98	*	0.96	0.94	0.99	*
**Age Group (years; ref. <11)**								
*11 to 20*	--	--	--		3.19	1.81	5.60	*
*21 to 30*	--	--	--		3.98	2.21	7.17	*
*31 to 40*	--	--	--		4.81	2.72	8.52	*
*41 to 50*	--	--	--		3.74	2.13	6.56	*
*51+*	--	--	--		4.55	2.00	10.35	*
**Screens or AC in home**								
*Yes*	--	--	--		0.35	0.22	0.55	*
**MODEL 8**								
*(Intercept)*	0.57	0.49	0.67		0.21	0.12	0.38	*
**Mobility variable**								
*Any time in location*: *work*	0.86	0.66	1.12		0.68	0.51	0.91	*
**Age Group (years; ref. <11)**								
*11 to 20*	--	--	--		3.17	1.82	5.54	*
*21 to 30*	--	--	--		4.30	2.38	7.76	*
*31 to 40*	--	--	--		5.53	3.10	9.85	*
*41 to 50*	--	--	--		4.21	2.39	7.41	*
*51+*	--	--	--		5.13	2.25	11.68	*
**AC in home**								
*Yes*	--	--	--		0.63	0.46	0.86	*
**MODEL 9**								
*(Intercept)*	0.53	0.44	0.63	*	0.17	0.09	0.33	*
**Mobility variable**								
*Hours in location*: *work*	0.998	0.992	1.004		0.9931	0.9865	0.9997	*
**Age Group (years; ref. <11)**								
*11 to 20*	--	--	--		3.61	1.94	6.75	*
*21 to 30*	--	--	--		4.95	2.55	9.61	*
*31 to 40*	--	--	--		6.68	3.51	12.71	*
*41 to 50*	--	--	--		5.06	2.68	9.57	*
*51+*	--	--	--		6.12	2.43	15.42	*
**AC in home**								
*Yes*	--	--	--		0.59	0.41	0.85	*
**MODEL 10**								
*(Intercept)*	0.66	0.55	0.78	*	0.25	0.14	0.44	*
**Mobility variable**								
*Any time in non-resident location inside with AC/Screens*	0.66	0.52	0.85	*	0.68	0.53	0.88	*
**Age Group (years; ref. <11)**								
*11 to 20*	--	--	--		3.25	1.85	5.68	*
*21 to 30*	--	--	--		3.88	2.17	6.95	*
*31 to 40*	--	--	--		4.74	2.69	8.36	*
*41 to 50*	--	--	--		3.56	2.04	6.22	*
*51+*	--	--	--		4.29	1.90	9.68	*
**AC in home**								
*Yes*	--	--	--		0.64	0.47	0.88	*
**MODEL 11**								
*(Intercept)*	0.59	0.49	0.72	*	0.19	0.10	0.37	*
**Mobility variable**								
*Hours in non-resident location inside with AC/screens*	0.992	0.986	0.998	*	0.992	0.986	0.998	*
**Age Group (years; ref. <11)**								
*11 to 20*	--	--	--		3.67	1.96	6.86	*
*21 to 30*	--	--	--		4.76	2.47	9.21	*
*31 to 40*	--	--	--		6.04	3.20	11.42	*
*41 to 50*	--	--	--		4.52	2.41	8.48	*
*51+*	--	--	--		5.47	2.18	13.72	*
**AC in home**								
*Yes*	--	--	--		0.60	0.41	0.86	*

Arboviral prevalence was associated with regularly spending time at work, but not with regularly visiting any other locations. After adjusting for age group and whether a person had AC in their home, any time spent outside the home in a work location was protective against prevalence of arbovirus (aOR: 0.68, 0.51–0.91) (Table 4, Model 8B). For each additional eight hours per week spent at work, there was an approximately 5% (0–10%) decrease in the odds of testing positive after adjusting for other variables (Table 4, Model 9B). Most work locations (83%) had AC or screens.

While visiting most specific location types did not have significant associations with test positivity, characteristics of those locations did. Similar to the workplace-specific finding, there was a general decreased prevalence among individuals who visited locations that were inside and had AC or screens, regardless of the type of location. That combination of location characteristics was associated with one of the largest decreases in arboviral prevalence of any factor we analyzed; any amount of time over 5 hours spent in a location that was inside with AC or screens was associated with an estimated 32% (12–47%) decrease in arboviral prevalence compared to individuals spending no time in locations with AC or screens after adjusting for age group and whether a person had AC in their home (Table 4, Model 10B). There was again a dose-response relationship between weekly hours spent in indoor locations with AC or screens and arboviral prevalence; for each additional eight hours per week spent in locations with these characteristics, there was an approximately 6% (1–10%) decrease in the odds of testing positive for ZIKV or CHIKV after adjusting for other variables (Table 4, Model 11B).

The intraclass correlation was similar across all mobility models, with a range of 0.57–0.59. In other words, slightly more than half of the overall variation in the outcome is explained by household clustering, while less than half was explained by these mobility variables.

## Discussion

Human mobility is a key component in the spread of arboviral diseases, often with a positive relationship where increased movement leads to increased incidence. We found a strong association between mobility and arboviral prevalence in our study, yet with a protective impact. More time spent outside of the home was associated with *decreased* prevalence of arbovirus among COPA study participants in southern Puerto Rico. Specifically, more time spent at work or indoor locations with AC or screens was associated with lower odds of testing positive for ZIKV or CHIKV.

Increased movement has been shown to spread arboviral disease by increasing an individual’s exposure to arboviruses in other locations [16–18]. However, in this population, the protective characteristics of locations visited appear to be the most important driver of the relationship between mobility and arboviral prevalence. This does not imply that mobility is not associated with exposure as some mobility is necessary to support geographical spread of arboviruses. However, it does indicate that not all mobility is the same, with elements like screens and AC providing protection in some locations. Our findings indicated similar levels of prevalence among individuals visiting locations without screens or AC to the prevalence of individuals spending time in and around their homes. These findings highlight the general importance of AC and screens, which are known to be protective against mosquitoes and mosquito-transmitted diseases such as dengue, Zika, and chikungunya [19,23,24]. Previous studies have shown that household AC and screens are important factors in determining arboviral risk; however, the results from this study further highlight their importance in non-residential locations where people visit. On the other hand, more time spent at home was associated with higher prevalence of arbovirus in this population, suggesting that the home may be the primary site of infection in this region. The main vector of CHIKV and ZIKV, Aedes aegypti, tends to feed and rest indoors. This anthropophilic nature further explains the finding that participants spending more time at home (where the vector feeds and rests) were more likely to have a positive arboviral result compared to the more mobile participants. These findings underscore the need for interventions focused around the home in Ponce, which are likely to be the most effective. In this study population, individuals with lower levels of mobility and who spent less time in air-conditioned and screened non-residence locations were more likely to have been infected by CHIKV or ZIKV. Individuals with lower mobility were more likely to be older, female, not employed full-time, and to have lower household income. Since individuals with these characteristics are less likely to spend time in locations with screens or AC outside the home, these sociodemographic factors are also likely risk factors for arboviral disease, potentially compounding other socioeconomic factors associated with risk like healthcare access and the ability to implement mosquito bite prevention measures in the home.

Individuals who spent time at work locations were also associated with lower prevalence of arbovirus. The finding that each additional 8 hours spent at work was associated with a 6% decrease in arboviral prevalence, while “very small” by most effect size standards [25,26], is not trivial when considering the burden imposed by infection with dengue. The majority of the work locations where participants spent time were also indoors with AC or screens. These results along with the fact that no other types of locations were associated with arboviral prevalence suggest that it is indeed the characteristic of AC or screens that is driving the association. While this study took place in southern Puerto Rico, the results can likely be generalized, as AC and screens have been shown to be protective for mosquito-borne disease in other settings [23,24]. The findings here indicate that this is important not only in the home, but also in other locations where people spend time. Policymakers can focus on requirements for AC and screens for businesses, schools, and other non-residential locations.

This study had some limitations. Precise, linked mobility data (e.g., from call detail records or smart phone data) were not available for this population, necessitating collection of mobility by surveys. Responses were subject to recall bias as individuals self-reported their mobility, or response bias for minors as their parents reported on their behalf. Participants may have changed mobility behavior throughout the study period due to external factors in the region such as earthquakes (December 2019–January 2020) or COVID-19 restrictions (March 2020 onward). To address these potential limitations, questions posed referred to mobility as visits to locations where participants spend at least 5 hours “during a normal week” to capture frequent, routine movement. However, this also means that irregular movement or regular movement for shorter durations, both of which could be important for arboviral disease transmission, may have been omitted.

Many regular weekly activities may take less than 5 hours (e.g., grocery shopping, running errands, attending church, visiting friends) and other irregular movement (e.g., visiting locations that are further away) are likely also important for arbovirus transmission. As a result, the mobility counts in this study are an underestimate of movement outside the home and cannot capture the risk associated with shorter-duration or irregular movement, both of which could be important for arboviral disease transmission.

We also were unable to characterize geographic variation in transmission. Most of the mobility reported in this population occurred within Ponce Municipality. We examined associations between prevalence and neighborhoods visited but were unable to detect specific geographic locations associated with different levels of arboviral prevalence.

Another potential limitation of this study was the different time scale of outcomes and predictors. The seroprevalence data were based on samples collected in Year 1 (May 2018–June 2019) and reflect infection over recent years (peak CHIKV transmission occurred in 2014 and ZIKV in 2016), while the full mobility questionnaire was administered in Year 2 (September 2019–November 2020) and aimed to capture current, “normal” mobility. Comparable data were not available for the same time period when exposure occurred. However, IgM levels wane over time, indicating that ZIKV levels in this study are likely an underestimate of all prior infections. We also compared a mobility-related question that was similar from Year 1 (“How much time do you spend in your house or in this community or urbanization?”) to Year 2 (“During a normal week, how many hours do you generally spend outside your home per day?”), and found strong correlation in responses across the two years. Additionally, while some people may change their mobility behavior over time due to shifts in lifestyle or aging, the change can happen in either direction (i.e., increased or decreased mobility) and we expect that the relationship is overall a representation of average mobility for the population. This limitation would likely produce a more conservative estimate than if the time scale had matched.

While information was collected for the presence of AC and screens in locations, no information was available about the quality of those characteristics. It is possible that some screens were not well-maintained. In this case, our estimates for the impact of screens on locations visited in relation to the prevalence of arbovirus were likely underestimates. Lastly, due to high correlation between many of the mobility variables, it was not possible to estimate the combined or adjusted association between multiple mobility variables and the prevalence of arboviral infection. Future studies could incorporate alternative mobility metrics (such as distance, trajectories, the quality of some of the collected variables, and bodily movements of participants), data sources (such as mobile phone data), and levels of data (at the neighborhood level or smaller) to supplement these findings.

Human mobility is an important contributor to infectious disease transmission dynamics. The finding that increased mobility to locations with AC or screens in this population in southern Puerto Rico was associated with decreased arboviral prevalence highlights the complex role of mobility, which is both necessary for disease spread and potentially protective in some situations. This study provides insights on both challenges and opportunities for arbovirus control, which should account for the role of human movement. The findings from this study also support the use of screens or AC in all locations, not just in the home, as important factors to decrease the risk for arbovirus transmission.

## Supporting information

S1 Fig Communities Organized to Prevent Arboviruses cohort (COPA) mobility questionnaire. (PDF)
