# Supplementary material for: Quantifying the relationship between arboviral infection prevalence and human mobility patterns among participants of the Communities Organized to Prevent Arboviruses cohort (COPA) in southern Puerto Rico
Source: PLoS Negl Trop Dis. 2023 Dec 15;17(12):e0011840. doi: 10.1371/journal.pntd.0011840 (PMC10756524; doi:10.1371/journal.pntd.0011840)
Supplement: S1 Fig — (PDF) [file pntd.0011840.s001.pdf]

Mobility

[All Participants]

Interviewer Name:

Interview Date: mm / dd / yyyy

Participant ID (**HHID P PID**): X X # # # # P # #

Interviewer, read the following:
In this section, we will ask you some questions related to places you spend time at during the week. We are asking these questions because, in the future, this information can help us know where people are getting sick with viruses transmitted by mosquitoes and to carry out activities to reduce mosquitoes in those areas. Remember that all this information is confidential, and only authorized study personnel will have access to it. We will not share your information with anyone else.

M0.1 During a normal week, how many hours do you generally spend **outside your home** per day (24 hrs)?

Monday: __________ hours
Tuesday: __________ hours
Wednesday: ______ hours
Thursday: _________ hours
Friday: _________ hours
Saturday: ______ hours
Sunday: ________ hours

M0.2 With the exception of your home, are there other places you spend **5 or more hours** a week (for example work, school, or another home)?

Show the list of places if necessary.

☐ Yes | ☐ No | ☐ NR

M0.3 Can you list the places you visit (where you might be 5 or more hours total per week)?

☐ Yes | ☐ No | ☐ NR

Select all that apply.

- ☐ Primary job
- ☐ Second job
- ☐ Third job
- ☐ University/college
- ☐ School
- ☐ Church
- ☐ Gym
- ☐ Child's daycare
- ☐ Other house
- ☐ Grocery store
- ☐ Any other store
- ☐ Sports activity 1
- ☐ Sports activity 2
- ☐ Sports activity 3
- ☐ Recreational activity 1
- ☐ Recreational activity 2
- ☐ Recreational activity 3
- ☐ Other location #1: ___________
- ☐ Other location #2: ___________
- ☐ Other location #3: ___________
- ☐ Other location #4: ___________

M1.1 **Location #1**

Select one.

- ☐ Primary job
- ☐ Second job
- ☐ Third job
- ☐ University/college
- ☐ School
- ☐ Church
- ☐ Gym
- ☐ Child's daycare
- ☐ Other house
- ☐ Grocery store
- ☐ Any other store
- ☐ Sports activity 1
- ☐ Sports activity 2
- ☐ Sports activity 3
- ☐ Recreational activity 1
- ☐ Recreational activity 2
- ☐ Recreational activity 3
- ☐ Other location #1: ___________
- ☐ Other location #2: ___________
- ☐ Other location #3: ___________
- ☐ Other location #4: ___________

M1.2 Do you visit this location year-round? ☐ Yes | ☐ No | ☐ NR

M1.2.1 Which months do you visit this location?

Select all that apply.

☐ January	☐ April	☐ July	☐ October
☐ February	☐ May	☐ August	☐ November
☐ March	☐ June	☐ September	☐ December

M1.3 Which days of the week do you go to this location?

Select all that apply.

☐ Monday	☐ Wednesday	☐ Friday	☐ Sunday	☐ Refuse
☐ Tuesday	☐ Thursday	☐ Saturday	☐ Don't know	

M1.4 How many hours do you generally spend at this location **each week**? ______ hours

M1.5 When you are at this location, are you indoors, outdoors, or both? ☐ Indoors ☐ Outside ☐ Both

M1.5.1 Does this location have **screens** in good condition on all the doors and windows? Read the options.

☐ Yes ☐ No ☐ On all that are opened ☐ Don't know ☐ NR

M1.5.2 Does this location use **air conditioning** when you are there? Read the options.

☐ Sometimes ☐ All the time ☐ No ☐ NR

M1.6 Can you find this place on a map? ☐ Yes | ☐ No | ☐ NR

Coordinates of the pin:

☐ Outside of Ponce

M1.7 Can you provide the address for this place?

☐ Yes | ☐ No

Physical address: ____

M1.8 In which area is this place located (sector, urb, barrio, community)? _____________________________________

M2.1 **Location #2**

Select one.

- ☐ Primary job
- ☐ Second job
- ☐ Third job
- ☐ University/college
- ☐ School
- ☐ Church
- ☐ Gym
- ☐ Child's daycare
- ☐ Other house
- ☐ Grocery store
- ☐ Any other store
- ☐ Sports activity 1
- ☐ Sports activity 2
- ☐ Sports activity 3
- ☐ Recreational activity 1
- ☐ Recreational activity 2
- ☐ Recreational activity 3
- ☐ Other location #1: ___________
- ☐ Other location #2: ___________
- ☐ Other location #3: ___________
- ☐ Other location #4: ___________

M2.2 Do you visit this location year-round? ☐ Yes | ☐ No | ☐ NR

M2.2.1 Which months do you visit this location?

Select all that apply.

☐ January	☐ April	☐ July	☐ October
☐ February	☐ May	☐ August	☐ November
☐ March	☐ June	☐ September	☐ December

M2.3 Which days of the week do you go to this location?

Select all that apply.

☐ Monday	☐ Wednesday	☐ Friday	☐ Sunday	☐ NR
☐ Tuesday	☐ Thursday	☐ Saturday	☐ Don't know	

M2.4 How many hours do you generally spend at this location **each week**? ______ hours

M2.5 When you are at this location, are you indoors, outdoors, or both? ☐ Indoors ☐ Outside ☐ Both

M2.5.1 Does this location have **screens** in good condition on all the doors and windows? Read the options.

☐ Yes ☐ No ☐ On all that are opened ☐ Don't know ☐ NR

M2.5.2 Does this location use **air conditioning** when you are there? Read the options.

☐ Sometimes ☐ All the time ☐ No ☐ NR

M2.6 Can you find this place on a map? ☐ Yes | ☐ No | ☐ NR

Coordinates of the pin:
☐ Outside of Ponce

M2.7 Can you provide the address for this place?

☐ Yes | ☐ No

Physical address: ______________________________

M2.8 In which area is this place located (sector, urb, barrio, community)? ______________________________

M3.1 **Location #3**

Select one.

☐ Primary job	☐ Sports activity 1
☐ Second job	☐ Sports activity 2
☐ Third job	☐ Sports activity 3
☐ University/college	☐ Recreational activity 1
☐ School	☐ Recreational activity 2
☐ Church	☐ Recreational activity 3
☐ Gym	☐ Other location #1: __________
☐ Child's daycare	☐ Other location #2: __________
☐ Other house	☐ Other location #3: __________
☐ Grocery store	☐ Other location #4: __________
☐ Any other store	

M3.2 Do you visit this location year-round?

☐ Yes | ☐ No | ☐ NR

M3.2.1 Which months do you visit this location?

Select all that apply.

☐ January	☐ April	☐ July	☐ October
☐ February	☐ May	☐ August	☐ November
☐ March	☐ June	☐ September	☐ December

M3.3 Which days of the week do you go to this location?

Select all that apply.

☐ Monday	☐ Wednesday	☐ Friday	☐ Sunday	☐ NR
☐ Tuesday	☐ Thursday	☐ Saturday	☐ Don't know	

M3.4 How many hours do you generally spend at this location **each week**? ______ hours

M3.5 When you are at this location, are you indoors, outdoors, or both? ☐ Indoors ☐ Outside ☐ Both

M3.5.1 Does this location have **screens** in good condition on all the doors and windows? Read the options.

☐ Yes ☐ No ☐ On all that are opened ☐ Don't know ☐ NR

M3.5.2 Does this location use **air conditioning** when you are there? Read the options.

☐ Sometimes ☐ All the time ☐ No ☐ NR

M3.6 Can you find this place on a map? ☐ Yes | ☐ No | ☐ NR

Coordinates of the pin:
☐ Outside of Ponce

M3.7 Can you provide the address for this place?

☐ Yes | ☐ No

Physical address: ______________________________

M3.8 In which area is this place located (sector, urb, barrio, community)? ______________________________

M4.1 **Location #4**

Select one.

☐ Primary job	☐ Sports activity 1
☐ Second job	☐ Sports activity 2
☐ Third job	☐ Sports activity 3
☐ University/college	☐ Recreational activity 1
☐ School	☐ Recreational activity 2
☐ Church	☐ Recreational activity 3
☐ Gym	☐ Other location #1: __________
☐ Child's daycare	☐ Other location #2: __________
☐ Other house	☐ Other location #3: __________
☐ Grocery store	☐ Other location #4: __________
☐ Any other store	

M4.2 Do you visit this location year-round? ☐ Yes | ☐ No | ☐ NR

M4.2.1 Which months do you visit this location?

Select all that apply.

☐ January	☐ April	☐ July	☐ October
☐ February	☐ May	☐ August	☐ November
☐ March	☐ June	☐ September	☐ December

M4.3 Which days of the week do you go to this location?

Select all that apply.

☐ Monday	☐ Wednesday	☐ Friday	☐ Sunday	☐ NR
☐ Tuesday	☐ Thursday	☐ Saturday	☐ Don't know	

M4.4 How many hours do you generally spend at this location **each week**? ______ hours

M4.5 When you are at this location, are you indoors, outdoors, or both? ☐ Indoors ☐ Outside ☐ Both

M4.5.1 Does this location have **screens** in good condition on all the doors and windows? Read the options.

☐ Yes ☐ No ☐ On all that are opened ☐ Don't know ☐ NR

M4.5.2 Does this location use **air conditioning** when you are there? Read the options.

☐ Sometimes ☐ All the time ☐ No ☐ NR

M4.6 Can you find this place on a map? ☐ Yes | ☐ No | ☐ NR

Coordinates of the pin:
☐ Outside of Ponce

M4.7 Can you provide the address for this place?

☐ Yes | ☐ No

Physical address: ______________________________

M4.8 In which area is this place located (sector, urb, barrio, community)? ______________________________

**Use an additional sheet if the participant has more than 4 key locations that they visit 5 or more house a week.*
